# Heads Down for Acute Stroke: Supine Position for Acute Large Vessel Occlusion in Ischemic Stroke

**DOI:** 10.1002/ccr3.71477

**Published:** 2025-12-30

**Authors:** Hassaan A. Khan, Emmanuel J. Thomas, Sufyan K. Zackariya, Kate M. Kelly, Saniya K. Zackariya, Zenia Qasim, Manaal Buchh, Ameera S. Khan, Hala K. Ansari, Holly P. Lankford, Leigh K. Van Ryn, Caroline C. Howell, Mahmoud D. Al‐Fadhl, Marie Nour Karam, Anthony V. Thomas, Rodolfo Gonzalez, Faisal S. Shariff, Adeela M. Alizai, Donald F. Zimmer, Mathew K. Marsee, Mark M. Walsh

**Affiliations:** ^1^ Department of Emergency and Internal Medicine Saint Joseph Regional Medical Center Mishawaka Indiana USA; ^2^ Indiana University School of Medicine – South Bend South Bend Indiana USA; ^3^ George Washington University School of Medicine Washington DC USA; ^4^ Department of Internal Medicine The University of Toledo Toledo Ohio USA; ^5^ Michiana Ophthalmology LLC Mishawaka Indiana USA; ^6^ Department of Medical Education Beacon Health System South Bend Indiana USA

**Keywords:** carotid artery thrombosis, collateral circulation, ischemic stroke, ophthalmic artery, stents

## Abstract

This case demonstrates that for large vessel occlusion strokes in the acute stage before surgical, pharmacologic, or radiologic intervention, supine positioning may be assumed because of the hemodynamic changes that can prevent and alleviate stroke, supporting the results of the ZerO Degree head positioning In Acute ischemiC stroke (ZODIAC) trial.

## 1 Introduction

It has been noted that the assumption of the sitting position for patients with large vessel occlusion (LVO) causes clinical deterioration during the hyperacute period with enough frequency to warrant a formal randomized controlled trial (RCT) comparing the benefits of the assumption of the supine position for those patients with LVO thrombotic strokes during the hyperacute period prior to stenting, thrombectomy, or thrombolysis [1, 2, 3]. Previous studies have failed to demonstrate an improvement with the supine position; however, these studies did not focus on LVO thrombotic strokes during the hyperacute period for those patients who were candidates for stenting, thrombectomy, or thrombolysis [4, 5, 6]. A major limitation of studies that failed to show an improvement with the supine position is the paucity of RCTs and the long period between the onset of symptoms and the assumption of the supine position in patients who have been studied [6]. We present an example of the unique applicability of the benefit of maintaining cerebral perfusion pressure during the hyperacute period of LVO. A 48‐year‐old hypertensive patient with a tobacco use history presented with a hyperacute internal carotid artery (ICA) occlusion on the left with right‐sided hemiparesis, hemianopsia, and aphasia, which occurred only in the sitting position and immediately disappeared with the assumption of the supine position. We describe the clinical course, radiologic findings, therapeutic intervention, and outcome to reinforce the importance of this simple procedure of assuming a supine position for this select group of patients and emphasize the cerebral hemodynamics associated with supine positioning to illustrate why it may be immediately effective in treating LVO stroke. We present this case report in accordance with the CARE reporting checklist with deidentified figures.

## 2 Case History/Examination

A 48‐year‐old male with a history of untreated hypertension and 30 pack‐year tobacco use experienced fluctuating right‐sided weakness and speech difficulty for 48 h. He took no medicines and had no prior history of hypercholesterolemia, coronary artery disease, or surgeries. He had not seen a primary care physician in three years. In the emergency department, the patient had right‐sided weakness, aphasia, and right hemianopsia. Vital signs were blood pressure 140/85 mmHg, pulse 80 bpm, respiration 16 breaths per minute, temperature 37.1°C, and oxygen saturation 97%. The neurologic exam revealed that in the sitting position, the patient had a right facial droop, expressive aphasia, 1/5 muscle strength in the right upper and right lower extremities with 1/4 brachioradial, biceps, and patellar reflexes and diminished pinprick sensation of the right upper and lower extremities. The right toe was downgoing with plantar stimulation. There was right‐sided homonymous hemianopsia. The National Institutes of Health Stroke Scale (NIHSS) was calculated at 15 points. Relevant laboratory data included a complete blood count with white blood cells 7400/mm^3^, hemoglobin 15.7 g/dL, hematocrit 47%, platelet count 360,000 cells/mm^3^, blood urea nitrogen 17 mg/dL, creatinine 1.0 mg/dL, potassium 4.1 mEq/L, sodium 138 mEq/L, chloride 97 mEq/L, bicarbonate 24 mEq/L, partial thromboplastin time (PTT) 27 s, prothrombin time 11.4 s, international normalized ratio 1.1, d‐Dimer 1.4 ng/mL, and Clauss fibrinogen 227 mg/dL. Cholesterol profile revealed. total cholesterol 200 mg/dL, low‐density lipoprotein cholesterol 170 mg/dL, high‐density lipoprotein cholesterol 22 mg/dL, triglycerides 160 mg/dL. Albumin, alanine aminotransferase, aspartate aminotransferase, alkaline phosphatase, and urinalysis revealed no abnormal results.

## 3 Differential Diagnosis

In the computed tomography (CT) scanner, the patient noted a complete resolution of symptoms when he assumed a supine position. However, with the assumption of a sitting position, the neurologic symptoms returned. This positional symptomatology caused the emergency physician to hypothesize that there may be position‐dependent filling of the intracerebral vessels during this acute ischemic event.

**FIGURE 1:**
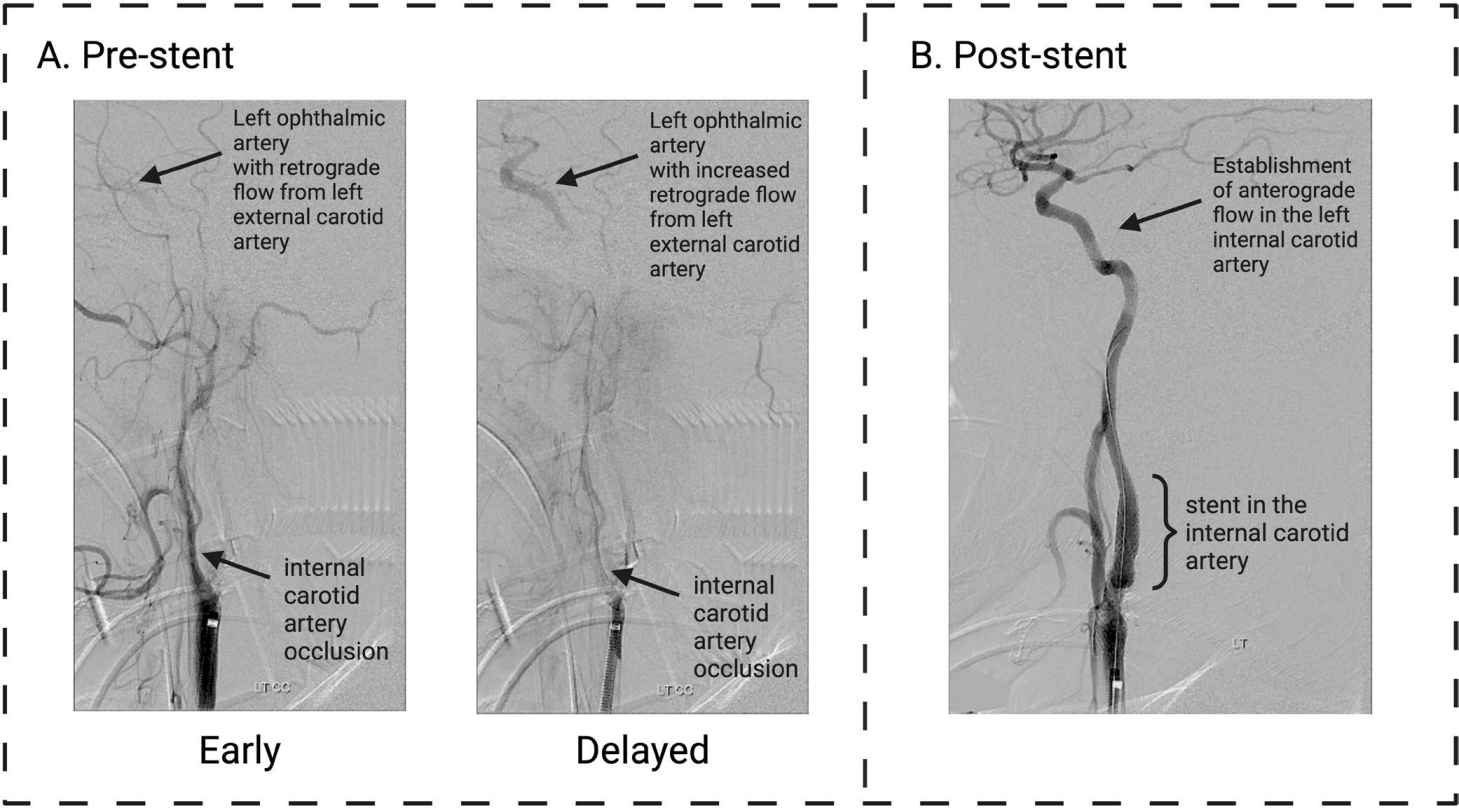
These images show the ophthalmic, external, and internal arteries of the patient before and after the addition of a stent. (A) Shows the computed tomography angiogram identifying the patient's left internal carotid artery occlusion with retrograde flow through the ophthalmic artery. Retrograde flow in the ophthalmic artery increased over time due to the occlusion in the internal carotid artery. (B) Demonstrates resolution of occlusion and return of normal antegrade flow in the internal carotid artery after the addition of a stent.

A CT scan of the brain without contrast confirmed the absence of an acute bleed. A CT angiogram (CTA, GE Revolution CT machine) revealed a cervical left ICA occlusion with retrograde flow through the left ophthalmic artery into the internal carotid terminus where it joins the left middle cerebral artery (MCA) (Figure 1A).

**FIGURE 2:**
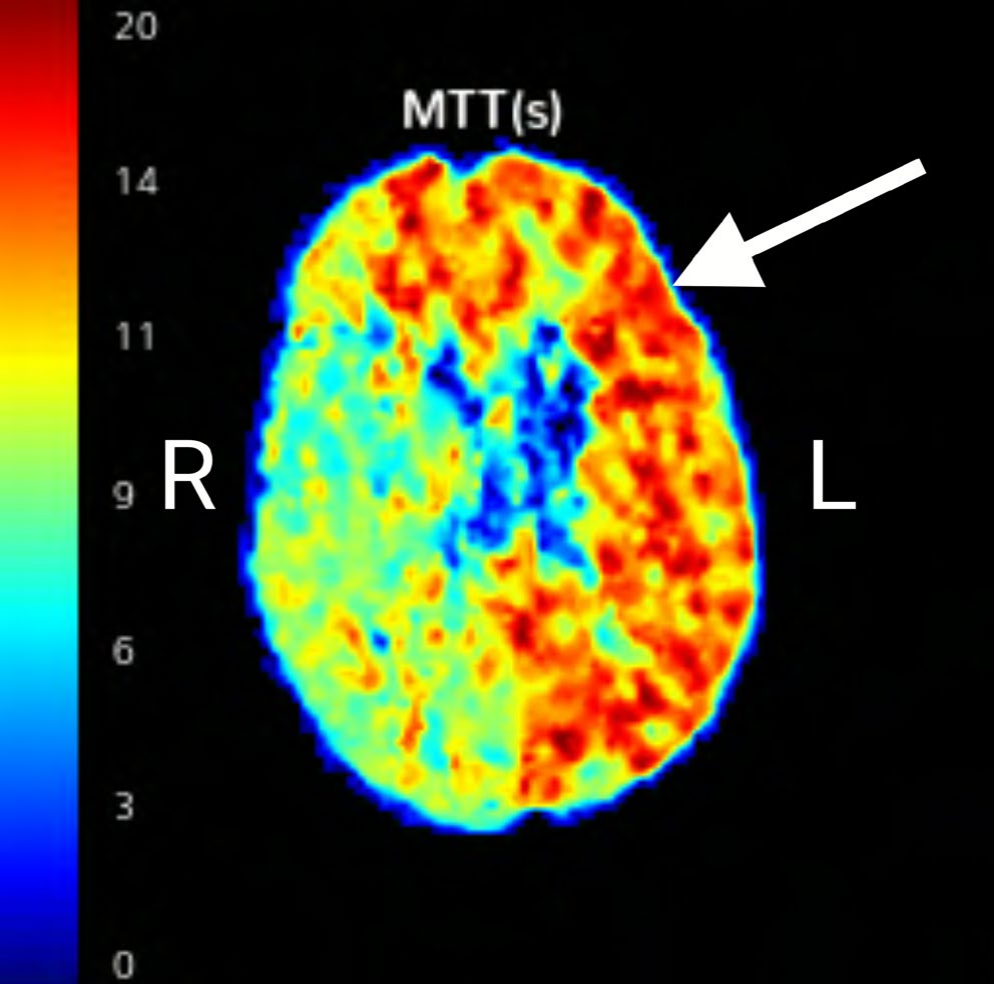
This computed tomography angiogram perfusion study shows increased mean transit time (MTT) in seconds on the left. At‐risk penumbra tissue in red (see arrow) involves the left (L) middle cerebral artery and bilateral anterior cerebral artery distribution involving the Circle of Willis and the retrograde ophthalmic artery. Normal flow is yellow‐green on the right (R). Penumbra is 12–14 s on the left or roughly 2.5 times longer than the contralateral normal side. When there is an arterial blockage to the brain, blood is slow to flow in and slow to wash out, so there is prolonged transit time. The left side of the brain is the abnormal side with prolonged transit time, and the right side is normal.

In addition, the CTA revealed severe atherosclerotic narrowing of the right vertebral artery due to atherosclerosis as well as a congenitally narrow right anterior cerebral artery. A CT cerebral perfusion analysis study revealed a large penumbra of at‐risk brain of the entire left cerebrum (Figure 2). Because of the tenuous blood flow to the entire left cerebrum, which was dependent on the hemodynamic improvement associated with the assumption of a supine position and which was supplied through the compensatory redirection of blood through the Circle of Willis (CoW) as well as the retrograde flow from the branches of the external carotid artery (ECA) through retrograde flow through the ophthalmic artery, it was decided to place an arterial stent. The patient was acutely symptomatic from occlusive cervical left ICA stenosis, possibly due to plaque rupture. This resulted in hypoperfusion to the left MCA and both anterior cerebral arteries (left A1 dominant, right A1 hypoplastic). Therefore, no thrombectomy was done. Transfemoral carotid stenting was successfully achieved with a 6–8 mm diameter by 40 mm long Abbott RX Acculink stent at the site of the occlusion in the cervical left carotid artery, which resulted in the return of blood flow to the left side of the brain.

The patient was given an initial bolus of 5000 units of intravenous heparin before the procedure. During the procedure, an initial maintenance rate of 1000 units/h was used and titrated to maintain a therapeutic activated PTT. At 24 h, the heparin was discontinued, and the patient was initiated on 81 mg aspirin and 75 mg clopidogrel by mouth daily. Post‐stenting angiography revealed complete reversal of the occlusion at the level of the ICA with resumption of normal flow direction through the ophthalmic artery (Figure 1B). Retrograde flow was noted around the occluded left ICA, revealing the major arterial conduits of compensatory reverse flow at the branches of the left ECA: facial, maxillary, and superficial temporal arteries and their tributaries (Figure 1).

## 4 Conclusion and Results

The patient remained in a supine position for 24 h and was discharged on day four with resolution of symptoms. Six months after the procedure, the patient remains on dual antiplatelet therapy and is symptom‐free. His medications now also include atorvastatin 40 mg/day orally and lisinopril 20 mg/day orally.

## 5 Discussion

**FIGURE 3:**
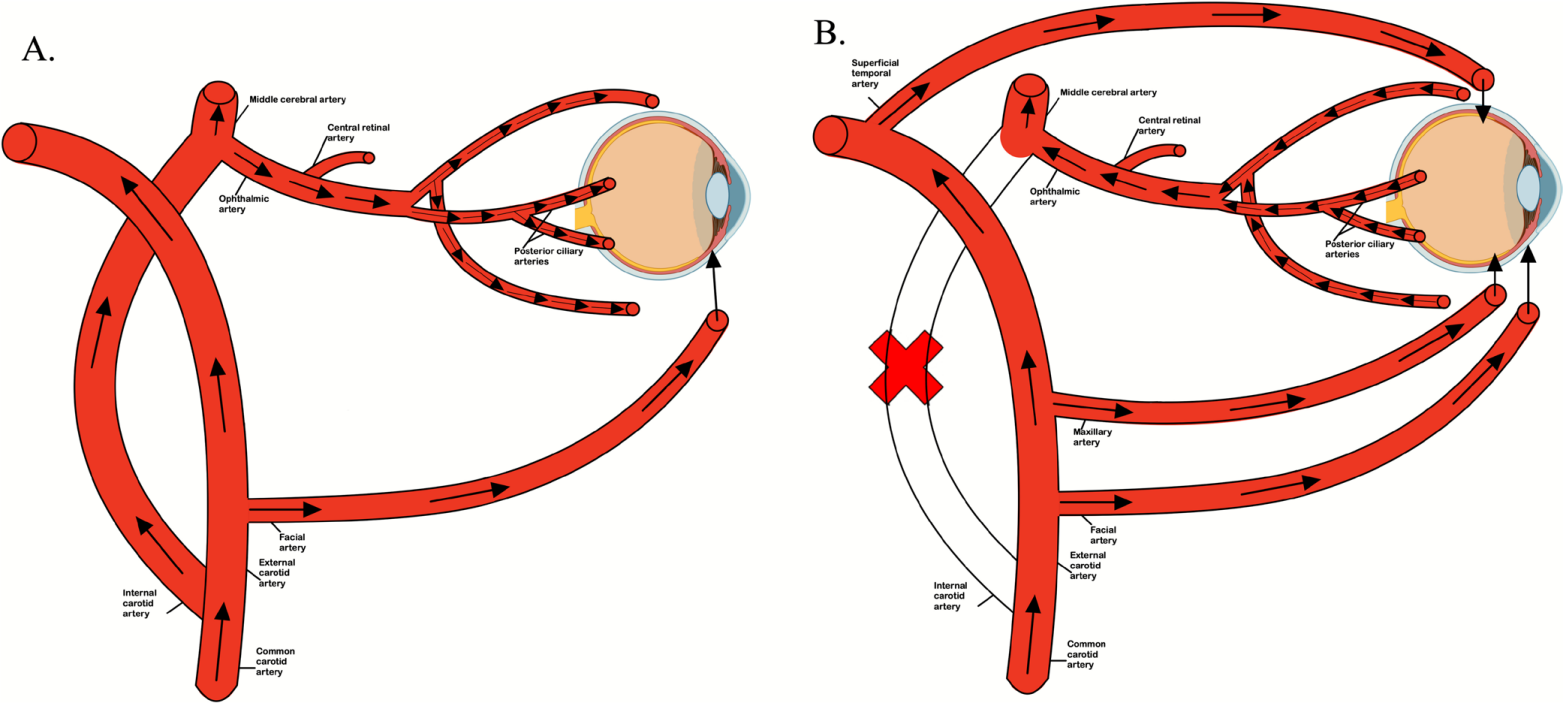
This figure illustrates blood supply to the eye with and without the presence of an occlusion in the internal carotid artery (ICA). (A) Shows blood flow to the eye under normal conditions. The eye is supplied by branches from the ophthalmic and facial arteries. (B) Shows blood flow to the eye when there is an occlusion in the ICA. The three major branches of the external carotid that supply retrograde flow to the ophthalmic artery are the facial, superficial temporal, and maxillary arteries. Normally, the superficial temporal and maxillary arteries do not supply the eye, but with occlusion of the ICA, both the maxillary and superficial temporal arteries may contribute to retrograde flow through the ophthalmic artery due to reduced pressure in the ophthalmic artery caused by the obstruction. Arrows from these vessels indicate perfusion through branches that anastomose around the eye to provide retrograde flow to the ophthalmic artery, which then supplies the middle cerebral artery at the bifurcation of the ICA. An angiosome is described as an anatomic unit that obtains its arterial blood supply from specific source arteries and is usually drained by corresponding veins. The supraorbital angiosome includes the skin of the forehead and scalp and the muscles of the forehead and is partially supplied by branches of the ophthalmic artery [7, 8, 9, 10, 11, 12].

Figure 3 demonstrates collateral flow through the facial, maxillary, and superficial temporal branches of the ECA through which the majority of flow to the eye supplies retrograde flow through the ophthalmic artery with occlusion of the left internal carotid [9, 10, 11, 12]. Normally, the maxillary artery does not directly supply the eye. However, it does supply blood flow to the structures around the eye. With ICA occlusion, the maxillary artery may contribute indirectly to the eye with resultant retrograde flow through the ophthalmic artery. This pattern of retrograde flow through the eye may be present in some patients with carotid occlusion and would be dependent on a patent ECA [10]. In cases where occlusion of the ICA is associated with retrograde flow through the ophthalmic artery, attention should be paid to the treatment not only of the carotid occlusion but also to potential atherosclerotic plaque removal in the ECA so as to maintain blood flow to the eye and prevent the development of ocular ischemia following the repair of the internal carotid occlusion. The changes in perfusion, pressure, collateral flow, and the activation of compensatory mechanisms directing flow through the CoW may be benefited by the hemodynamic and neurologic changes, such as improving the deficits in motor control, visual processing, and speech caused by reduced perfusion in the primary motor cortex, visual pathway, and Broca's area, brought about by the supine position since these patients are dependent on rapidly changing collateral blood flow to avoid permanent neurologic damage. Supine position during LVO exhibits physiologic changes to the specific neural substrates most affected by changes in cerebral perfusion. As noted above, the primary motor cortex, Broca's area, and visual pathways are particularly vulnerable in the context of LVO, and supine positioning may differentially impact these substrates. The anatomic and pathophysiologic discussion of retrograde flow to the ophthalmic artery and the changes noted in perfusion, pressure, and collateral flow provide valuable insight and further contextualize the clinical relevance of the supine position for these patients [1, 13, 14, 15, 16, 17, 18].

The ZerO Degree head positioning In Acute ischemiC stroke (ZODIAC) trial recently addressed positional exacerbation of cerebral blood flow in the region of the penumbra of the ischemic territory for patients with LVO strokes. This study explored the benefits of the patient's physical position in the event of an LVO ischemic stroke [2, 3]. The researchers proposed that laying stroke patients flat before thrombectomy and/or stent placement improved neurological function significantly compared to patients whose heads were raised at a 30‐degree angle. They found that this positioning was safe, and it enhanced blood flow to the brain, potentially reducing the risk of neurological deficits and disability [2, 3]. Of importance with the small ZODIAC trial is that the number needed to harm was 1.88 for those patients who did not assume a supine position with acute LVO strokes and 2.48 for a deterioration of at least 4 on the NIHSS when sitting versus supine positioning in the preprocedural period during the hyperacute presentation of these LVO strokes [2, 3].

However, previous large RCTs have evaluated the effect of supine and sitting position during the acute stroke period and failed to find a beneficial effect of supine positioning [4, 5, 6]. This suggests that the assumption of supine positioning may not be as necessary as the ZODIAC trial suggests and that the results of the ZODIAC trial may be more useful for hypothesis generation than for clinical practice. However, the duration of stroke symptoms for the patients in these previous large RCTs before the assumption of a supine or sitting position varied greatly among patients and had a median of 14 h in one study; additionally, that study included small infarcts in which a positional change would not be as likely to cause any meaningful effects [4, 5, 6]. This indicates that further validation of the ZODIAC trial may still be warranted.

Regardless, the ZODIAC trial's results are still limited in clinical applicability since it was a small trial that had a primary negative endpoint of a deterioration of at least 2 on the NIHSS, which has not been shown to be significantly indicative of worse outcomes at 90 days. The American Heart Association (AHA) guidelines still do not recommend supine positioning for all patients with hyperacute strokes awaiting surgical or radiological intervention. Today, there remains an evolving analysis with a proposal for a more rigorous and comprehensive RCT to test the hypothesis of the ZODIAC trial and the efficacy of supine positioning in preventing long‐term negative outcomes for patients with severe LVO, while excluding patients with small infarcts who may skew the results [4, 5]. Supine positioning has been associated with loss of airway patency and increased risk for aspiration pneumonia (and other risks including worsening of brain edema, heart failure leading to hypoxia and intolerance), and the recommendation for the supine position for patients with reduced consciousness remains in a state of evolution which may be due to the selection bias of the Head Position in Stroke Trial (HeadPoST) who were more vigorously screened for dysphagia resulting in only 3% occurrence of aspiration pneumonia in that study [5, 16, 17, 19]. Reduced consciousness, emergent intubation, or vomiting should be exclusionary criteria for the assumption of supine positioning [3]. In addition, the effect of position on cerebral blood flow is not uniformly agreed upon. One systematic review demonstrated that intracranial pressure reduced with head elevation, but the effects of head elevation on cerebral perfusion pressure were varied [4, 5, 20]. Transcranial Doppler (TCD) using maximum flow velocity and pulsatility index has shown that flat positioning improves blood flow velocity in acute ischemic stroke [21]. TCD studies have furnished pathophysiologic and hemodynamic justification that supine positioning may prevent early neurologic deterioration in patients with LVO [21].

This case report highlights an important presentation of acute ischemic stroke with symptoms dependent on patient positioning. Recognizing the importance of collateral flow and improvement of flow made possible by the CoW, as well as the delicate nature of this compensation in patients in the hyperacute stages of a LVO in the supine position, is crucial to improving outcomes for patients with the same condition. Limitations to the applicability of this case report to clinical practice include that this is a single patient's case, and there has been no long‐term imaging follow‐up. The patient was reluctant to engage in further imaging, since he remained asymptomatic 1.5 years after the event. Future studies that include cases such as this one will determine the applicability of the ZODIAC trial. In addition, such studies will help define the true incidence of benefit of supine positioning for patients presenting with LVO strokes and determine if the benefits of the supine position can be replicated in a large cohort. Nevertheless, there are challenges in performing larger RCTs on this group of patients since participation may be inhibited by emergency physicians who may fail to recognize the need for the immediate assumption of the supine position. However, such a simple procedure seems a worthwhile inclusion into stroke protocols and has recently been recommended by the AHA committee on stroke management [3].

## Author Contributions

**Hassaan A. Khan:** conceptualization, visualization, writing – original draft, writing – review and editing. **Emmanuel J. Thomas:** conceptualization, visualization, writing – original draft, writing – review and editing. **Sufyan K. Zackariya:** conceptualization, writing – original draft, writing – review and editing. **Kate M. Kelly:** writing – original draft, writing – review and editing. **Saniya K. Zackariya:** conceptualization, writing – original draft, writing – review and editing. **Zenia Qasim:** writing – original draft, writing – review and editing. **Manaal Buchh:** conceptualization, writing – original draft, writing – review and editing. **Ameera S. Khan:** conceptualization, writing – original draft, writing – review and editing. **Hala K. Ansari:** conceptualization, writing – original draft, writing – review and editing. **Holly P. Lankford:** visualization, writing – original draft, writing – review and editing. **Leigh K. Van Ryn:** writing – original draft, writing – review and editing. **Caroline C. Howell:** writing – original draft, writing – review and editing. **Mahmoud D. Al‐Fadhl:** visualization, writing – original draft, writing – review and editing. **Marie Nour Karam:** writing – original draft, writing – review and editing. **Anthony V. Thomas:** writing – original draft, writing – review and editing. **Rodolfo Gonzalez:** writing – original draft, writing – review and editing. **Faisal S. Shariff:** writing – original draft, writing – review and editing. **Adeela M. Alizai:** writing – original draft, writing – review and editing. **Donald F. Zimmer:** writing – original draft, writing – review and editing. **Mathew K. Marsee:** writing – original draft, writing – review and editing. **Mark M. Walsh:** conceptualization, project administration, visualization, writing – original draft, writing – review and editing.

## Disclosure

The authors have completed the CARE reporting checklist.

## Ethics Statement

The authors are accountable for all aspects of the work in ensuring that questions related to the accuracy or integrity of any part of the work are appropriately investigated and resolved. All procedures performed in this study were in accordance with the ethical standards of the institutional and/or national research committee(s) and with the Helsinki Declaration (as revised in 2013). Written informed consent for publication of this case report and accompanying image was obtained from the patient.

## Conflicts of Interest

The authors declare no conflicts of interest.

## Data Availability

Data sharing is not applicable to this article as no new data were created or analyzed in this study.
